# Preparing Offspring for a Dangerous World: Potential Costs of Being Wrong

**DOI:** 10.1371/journal.pone.0048840

**Published:** 2012-11-07

**Authors:** Michael Coslovsky, Heinz Richner

**Affiliations:** Institute of Ecology and Evolution, University of Bern, Bern, Switzerland; Estacion Experimental de Zonas Áridas (CSIC), Spain

## Abstract

Adaptive maternal responses to stressful environments before young are born can follow two non-exclusive pathways: either the mother reduces current investment in favor of future investment, or influences offspring growth and development in order to fit offspring phenotype to the stressful environment. Inducing such developmental cues, however, may be risky if the environment changes meanwhile, resulting in maladapted offspring. Here we test the effects of a predator-induced maternal effect in a predator-free postnatal environment. We manipulated perceived predation-risk for breeding female great tits by exposing them to stuffed models of either a predatory bird or a non-predatory control. Offspring were raised either in an environment matching the maternal one by exchanging whole broods within a maternal treatment group, or in a mismatching environment by exchanging broods among the maternal treatments. Offspring growth depended on the matching of the two environments. While for offspring originating from control treated mothers environmental mismatch did not significantly change growth, offspring of mothers under increased perceived predation risk grew faster and larger in matching conditions. Offspring of predator treated mothers fledged about one day later when growing under mismatching conditions. This suggests costs paid by the offspring if mothers predict environmental conditions wrongly.

## Introduction

Predation is a major selective force for the evolution of life-history traits and anti-predator behaviors in parents and offspring, e.g. [1], [2]. Young animals are particularly vulnerable to predation due to their smaller size and immature senses and responses, and due to their dependence, in many species, on the parents for feeding and protection [3]. This affects the evolution of behaviors such as parental guarding [1], food provisioning [3], and of life-history traits such as age of maturation [3], [4].

Females can show a variety of responses to predation risk during reproduction [2], which entail different consequences for females or offspring. A mother may simply reduce investment in current reproduction in favor of future reproduction, which may maximize her own reproductive success at the expense of the current offspring. Alternatively, a female exposed to predation during prenatal stages such as ovulation, pregnancy or incubation may change egg composition, gestation or incubation behaviors to increase the fit of offspring to a given environment, a response commonly termed a maternal effect [5], [6]. If induced by predation, a maternal effect may change the allocation of resources within individual offspring, which would be observed as a change in offspring growth rate and/or survival for example [7], [8], [9].

Whether an observed maternal effect is adaptive may be difficult to determine. For example, increased levels of corticosterone (CORT, the stress hormone in birds) in eggs, possibly occurring after exposure to predation risk [10], may reduce hatching mass and nestling growth [10], [11] thus appearing maladaptive since nestling size and mass at fledging are known to be good predictors of winter survival and breeding in following years, e.g. [12]. Similarly, hatchability of eggs with increased levels of CORT is reduced, e.g. [10], [13]. These responses may be adaptive, however, if lower nestling mass increases maneuverability by way of reducing wing loading in favor of faster escape, resulting in a phenotype preferred in a post-fledging predator rich environment [14], [15], or, for females, if a reduction in current investment enhances future reproduction [11], [16].

Two recent experiments indeed suggest that predator-induced maternal effects may mediate investment in traits favoring faster escape in birds. Starlings hatching from eggs injected with CORT performed better in flight performance trials, and had lower wing loadings and more mature flight muscles than controls [15]. Similarly, offspring of great tit mothers exposed to model predators during ovulation were lighter and showed accelerated wing growth close to fledging [7].

Determining whether an observed change in offspring growth is adaptive requires also determining whose fitness is directly maximized – that of the mother, that of the offspring, or both. Mothers may actually increase lifetime reproductive success by reducing the investment into the current brood, which is obviously against the interest of current offspring [6]. An adaptive maternal effect should increase offspring fitness by a preparation for the maternal environment, which then requires the maternal pre-breeding environment be similar or ‘match’ the offspring growth and living environments [6], [16], [17]. This would occur when environmental conditions do not change much with respect to the length of the species’ generation cycle. Unpredicted changes in the environment may either worsen conditions for the offspring and/or breeders or improve them. In either case – not preparing offspring to bad conditions arriving, or preparing them to bad conditions that are not fulfilled – a ‘mismatch’ occurs between the maternal environment and that in which offspring live. Such a ‘mismatch’ may cause an induced maternal effect to be inefficient or even to become detrimental for the offspring [6], [16], [18], [19].

Here we tested the consequences of matching and mismatching of the maternal pre-laying and the postnatal environments for offspring growth. Starting before egg-laying, we experimentally manipulated perceived predation risk by exposing female great tits (*Parus major*) to stuffed models and calls of a typical local avian predator to create high perceived predation risk areas, and to models and calls of a non-predatory local bird in control areas. Exchanging broods after hatching between either ‘matching’ or non-matching (‘mismatch’) environments, created a full-factorial design with two factors: the maternal prenatal treatment (increased perceived predation risk vs. control) and the matching of the prenatal to the postnatal environment (match vs. mismatch) as a second treatment. Following nestling growth in this design allowed us to assess the importance of the maternal effect for mothers and offspring, and the relevance of the matching of prenatal maternal and postnatal environments. We predicted, following previous results [7], that offspring of mothers exposed to increased predation risk would be smaller and lighter than those of control mothers, but that they would show adaptations to predator rich environments such as increased wing growth. We further predicted that these effects and their strength will depend on the matching of the prenatal maternal and the postnatal environments: e.g. size and mass of offspring from mothers exposed to predation risk before and during egg laying would be even smaller, and wing growth possibly further increased under the matching treatment than under mismatching conditions. The largest differences in phenotypic traits was predicted to occur among the two matched treatments, whereas the two mismatched groups were predicted to show intermediate results. However, since the specifics of a ‘good phenotype’ under a specific risk of predation are not fully known, and since other environmental factors also have an effect, it is difficult to make precise predictions.

## Materials and Methods

### Ethics Statement

This study was approved by the Ethical Committee of the Agricultural Office of the Canton Bern, Switzerland (experimentation permit 117/07 to MC) and the Federal Agency for Environment of the Canton Bern, Switzerland (ringing permit 2736).

### Experimental Design

The study was carried out during spring 2010 in a forest near Bern, Switzerland (46°57’N, 7°24’E). About 250 pairs of great tits (*Parus major*) freely breed in nest boxes hanging in the forest. We divided the forest into 22 plots (14–15 nest boxes each) approximately two great tit territories apart, ca. 120 m [20], [21], in order to reduce treatment effects between neighboring plots. We closely monitored nest boxes in order to determine the start of nest-building, egg-laying, and incubation and hatching dates. After the last measurement day we checked nests every afternoon for fledging.

### Manipulation of Predation Risk

In order to increase perceived predation risk in half the plots of the forest (‘predator’ treatment), we displayed stuffed models of sparrowhawks (*Accipiter nisus*) while playing sparrowhawk calls from portable loudspeakers in central locations in each plot. The sparrowhawk is a common predator of great tits, and breeds during spring and early summer when the tits fledge [20], [21]. In the remaining plots we created a ‘control’ treatment by displaying song thrush models (*Turdus philomelos*) and playing song thrush songs. Song thrushes differ from great tits in nest type and foraging preference, and thus the temporary addition of two individual dummy birds is unlikely to influence perceived interspecific competition. This choice of a control species is preferred over random sounds and models as it avoids potential stress or other responses to unfamiliar or novel noises, which could also induce some kind of maternal effect and thus confound our results. Both bird species used for the simulation treatments are common resident species in the forest.

Two stuffed birds of either the predator or control species were placed in each plot for 1.5–2 h every day in the morning or in the evening, alternated sequentially. Sounds were played from portable loudspeakers (FoxPro NX3 game caller, FoxPro, USA, http://www.gofoxpro.com/) placed below the models while displayed. We displayed the models on wooden poles placed in eight central locations of each plot before the breeding season started. The poles were used sequentially so that a simulation was performed on each pole once every four days. In order to reduce potential effects of the treatments on the choice of territories by birds, which could confound results, and to randomize treatments according to the timing of reproduction, which can correlate with plot quality and conditions as well as with bird quality [22], simulations in a plot started when either (1) five nests reached a stage indicating territory use and that the nests are likely to be finished (nest box floor completely covered with 2 cm of fresh nest material), or (2) at least one nest in the plot reached an advanced stage before laying eggs (egg cup clearly visible, often padded with fur). The treatment for the first plot to start simulations was decided by rolling a die. Subsequent plots were assigned to treatments alternately by order of reaching one of the above stages, or by rolling a die if more than one plot.

Mean laying date did not differ among the two treatments (ANOVA: predator treatment *F*_1,123_ = 0.616, *p* = 0.434; details in supplemented material, Table S1), which suggests proper randomization of treatments over the season. Females of both treatment groups were exposed to the treatments for a similar number of days before starting to lay eggs (ANOVA: predator treatment: *F*_1,123_ = 1.781, *p* = 0.185; details in supplemented material, Table S1). On the third incubation day we weighed and counted the eggs of each clutch (±0.1 g) for calculation of mean egg mass.

### Brood Exchange and Nestling Measurements

To disentangle the maternal effects from the effects of the postnatal rearing environment we exchanged whole broods between nests. In order to test the effect of the matching or mismatching of environmental conditions between the prenatal maternal environment and the postnatal environment, we used the exchange as a treatment with two levels, exchanging either within (‘match’) or among (‘mismatch’) predator treatments. We exchanged broods with the same number of nestlings (±1) on the second day after hatching of the first nestling (hatching  =  nest day 0). The specific treatment (match vs. mismatch) was determined by rolling a die when more than one possibility arose. Before exchanging broods we individually weighed nestlings using a portable scale (±0.1 g) and marked them by plucking specific combinations of tuft feathers. During the transfer nestlings were kept in padded boxes, with heating bags to keep nestlings warm. Altogether the transfer process took one hour at the most, i.e. each nestling spent a maximum of 30 minutes outside of a nest box. We blocked the entrance hole of the nest boxes from which nestlings were removed with a piece of cloth to prevent parents from potentially deserting the nest after finding an empty nest box.

After brood exchange there were 32 and 31 nests originating from the maternal control treatment, under the matching and mismatching treatments respectively. The same numbers were true for nests originating from the maternal predator treatment.

Nestlings were measured again 8 and 15 days after hatching of the first nestling. On these days, in addition to mass, we measured nestling tarsus length (±0.05 mm), and wing length (±0.5 mm). On day 8 we sampled nestling blood for molecular sexing [23]. Sex ratio did not differ between the treatment groups (binomial regression; predator treatment: χ^2^ = 0.123, *p* = 0.726; match: χ^2^ = 0.258, *p* = 0.611; predator x match interaction: χ^2^ = 0.265, *p* = 0.607).

### Parental Feeding Behavior

Feeding rate (number of feeding events per hour) was assessed by recording nest entries for 1.5 h eight days after hatching. A digital camcorder was placed on the ground or on a branch below the nest aiming at the entrance hole. Recordings were performed in the morning (between 07∶00 and 11∶00). The first half hour of each video was discarded since the placing of the camera could potentially cause a temporary disturbance. We assessed feeding rate separately for male and female parents if possible. When video quality did not allow determining sex, we counted total number of parental entries, which corresponds to food provisioning during this nestling period.

### Statistical Procedures

Statistical analyses were done using R 2.12.0 [24]. To test the effect of our treatments on nest desertion, we defined three categories: nests that were deserted before eggs were laid, nests that were deserted after some eggs had been laid, and nests that were not deserted. We used a Chi-square test of independence between desertion rate (frequency in each category) and the two maternal treatments.

The time period where females were exposed to the treatment before starting to lay eggs was highly correlated with both the date on which the first egg was laid (*r* = 0.816, *p*<0.001), and the date of hatching (*r* = 0.697, *p*<0.001). This prevented us from testing these two factors independently within our statistical models. Since laying and hatching dates convey more biological information than the time span between treatment and hatching, we chose to use the former in the respective models.

Mean egg mass per nest was analyzed using a linear model controlling for laying-date of the first egg. We tested hatchability using a binomial generalized linear model (GLM), correcting standard errors using a quasi-GLM model due to overdispersion (residual deviance 485.82 on 203 degrees of freedom).

We tested whether the number of fledglings from each nest depended on the two treatments using a Poisson GLM. We included hatching date as a covariable, as well as brood size on day 2 (N2) and N2^2^, suggesting a brood size that maximizes fledging numbers, since it improved model fit and proved significant. Both treatments (control/predator and match/mismatch) were included as categorical factors together with the interaction between them.

Nestling morphological traits were analyzed separately using linear mixed effects models, utilizing the R package nlme [25]. Possible random effects of the plots of origin and of rearing were discounted since they proved not significant by Likelihood Ratio tests of nested models using the lme4 package [26], and also because including crossed random effects would render the computation much more complex. In addition, since the nestlings in every two nests which were exchanged could be seen as non-independent data points (within an ‘exchange-pair’), we tested the significance of ‘exchange-pair’ as a random effect. Since it was non-significant in all the models, and since ‘exchange-pair’ conveys hardly any biological information, we discounted also this random effect from the final models. In the models we used the varIdent variance function allowing different variances for the different treatment levels or, for mass analysis, for different ages [27]. For nestling mass we included an autoregressive correlation structure of order 1 [27].

As fixed effects in models of nestling morphological traits we included brood size on day 2 after hatching, hatching date, nestling sex, and the two treatments. Nestling age was included as a fixed factor to account for the repeated measurements. In the model of nestling mass we treated age as an ordered categorical factor to ease interpretation of different growth periods while testing for a quadratic relationship. Interactions between nestling age and the two treatments representing differences in growth rate were also included in models. To account for non-independence of nestlings from the same nest of origin, growing in the same foster nest, we included the nest of origin as a random effect in the models (since we exchanged whole broods, nest of origin accounts for the rearing environment as well). In repeated measurement models nestling identity was included as a random factor (nested within Nest of Origin). When necessary, we performed Tukey adjusted *post hoc* tests using the glht function from R package multcomp [28].

We compared feeding rates between the treatments using a negative-binomial GLM using the glm.nb function from the R package MASS [29] due to overdispersion when using a Poisson distribution for count data (residual deviance 931.42 on 99 degrees of freedom).

In all statistical models we removed interactions when non-significant (*p*>0.1) in order to allow the interpretation of main effects [30].

## Results

Maternal treatments were successful and did not increase the probability of nest desertion (χ^2^ = 0.284, *p* = 0.594). There was no difference in incubation duration for mothers of the different predator treatments, controlling for laying date (online material; laying date: *F*_1,121_ = 0.150, *p* = 0.700; treatment: *F*_1,121_ = 0.138, *p* = 0.712).

Clutch size did not differ between the maternal treatments when controlling for laying date (online material; laying date: *F*_1,234_ = 1.030, *p* = 0.573; treatment: *F*_1,234_ = 0.065, *p* = 0.888). Mean egg mass did not differ between the maternal treatment groups either (online material; *F*_1,213_ = 2.487, *p* = 0.116). Egg mass was related to clutch size, with eggs in larger clutches being increasingly lighter (estimate±SE: −0.011±0.005; *F*_1,213_ = 5.985, *p* = 0.015), but no significant interaction between clutch size and predator treatment (*F*_1,212_ = 0.116, *p* = 0.733). The probability to hatch was smaller in larger clutches, but did not differ between the treatment groups (Table 1).

**Table 1:** Summaries for hatching and fledging probability GLMs.

Model	Variable	Estimate (SE)	z	P
Hatchingprobability	Intercept	2.657 (0.547)	––	––
	Laying date	0.013 (0.017)	0.757	0.450
	Clutch size	−0.123 (0.060)	−2.063	0.040
	Predator treatment	0.219 (0.208)	1.049	0.295
Number fledged	Intercept	1.618 (0.076)	––	––
	N2 (centered)	0.072 (0.033)	2.157	0.031
	N2 (centered) ^2^	−0.044 (0.018)	−2.510	0.010
	Hatching date	0.026 (0.013)	2.567	0.010
	Predator treatment	0.026 (0.081)	0.321	0.748
	Match treatment	0.095 (0.082)	1.152	0.249
	Predator x Match	−0.010 (0.161)	−0.060	0.952

Coefficients are untransformed and stem from a GLM with binomial (hatching probability) and Poisson (number fledged) errors. Laying and hatching are centered for ease of interpretation. Reference level for all models is a nest from the prenatal control-match treatments. Values for non-significant interactions are just before removal from the model. N2 =  number of nestlings on day 2 after first hatch. SE  =  Standard error.

Fledgling numbers were not significantly affected by the treatments or their interaction (Table 1) although fledgling numbers slightly increased with the season’s progress and showed a maximum for nests with about 8 nestlings at hatching (Table 1).

**Figure 1:**
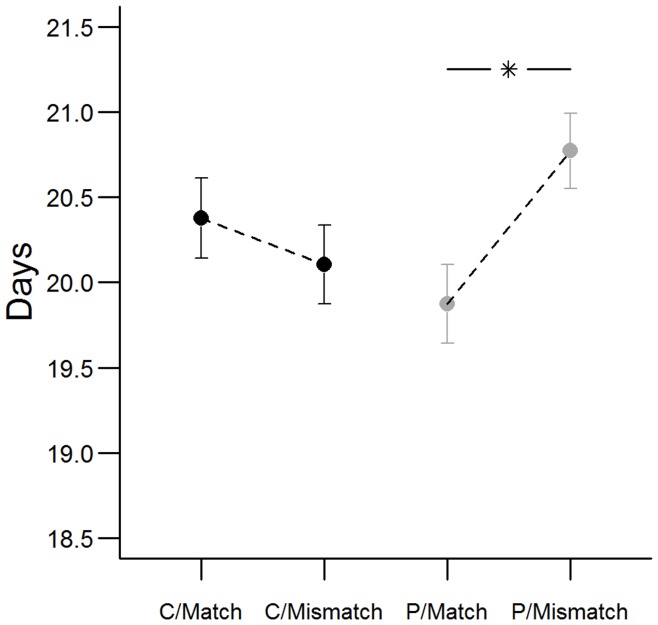
Fledging Age. Fledging age (model estimations of mean ± SE) for offspring of mothers exposed to either control birds (C) or to predatory birds (P) before and during egg-laying, raised either under matching or mismatching conditions. Asterisk represents significant difference (*p*<0.05).

There was a significant interaction between the two treatments explaining fledging age (Table 2; Fig. 1). Offspring of mothers exposed to prenatal increased predation risk fledged on average 0.89 days later when raised in a mismatching environment than in a matching one (Tukey adj. *post hoc*: *p* = 0.038). In contrast, offspring of mothers exposed to the prenatal control treatment did not differ significantly in fledging time if raised in matching versus mismatching environments (Tukey adj. *post hoc*: *p* = 0.822).

**Table 2:** ANOVA table for fledging age.

Variable	Estimate (SE)	df	*F*	*p*
Intercept	20.377 (0.236)	–	–	–
Brood size on day 2	0.218 (0.091)	1,105	5.813	0.018
Hatching date	−0.051 (0.029)	1,105	3.050	0.084
Predator treatment	−0.502 (0.329)	1,105	0.197	0.658
Match treatment	−0.273 (0.331)	1,105	1.946	0.166
Predator x Match	1.170 (0.459)	1,105	6.500	0.012

Reference level for treatment coefficients is prenatal maternal control treatment and matching environment. Brood size and hatching date centred.

**Figure 2:**
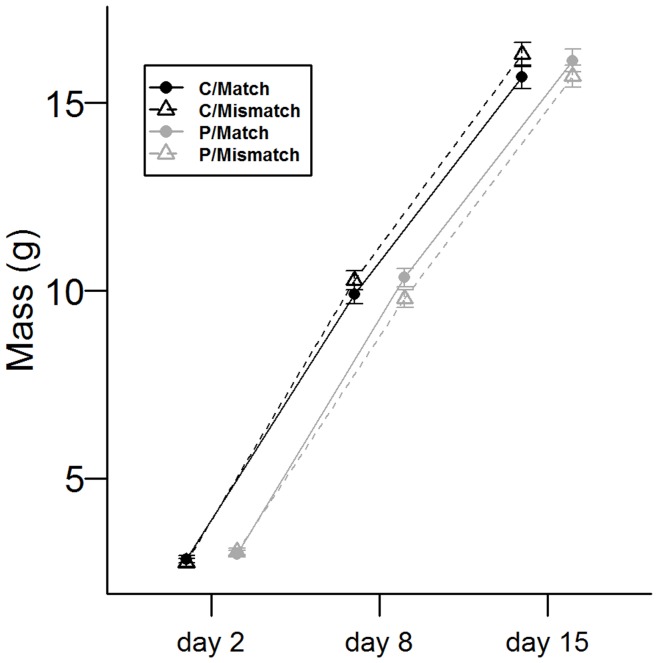
Mass growth curves. Nestling mass on three measurement days (Mixed Effects Model estimations of mean ± SE). The shape of growth curves differed significantly according to the interaction between the treatments. When mothers were exposed to predation risk before or during ovulation, growth depended on offspring environment. When growing with predation risk, i.e. a matching environment, early growth rate increased (steeper slope between days 2–8) compared to mismatching conditions. Under mismatching conditions, the fast mass gain, as well as reaching asymptotic mass, was postponed. C = mothers exposed to control treatment; P = mothers exposed to predator treatment.

Growth trajectories of nestling body mass depended on the interaction between the maternal treatment and the match treatment (significant Age x Predator x Match interaction; Table 3; Fig. 2). In the early growth phase, i.e. between days 2–8, nestlings of mothers exposed to prenatal high predation risk gained body mass faster under matching conditions than under mismatching conditions (Fig. 2; [coef. ± SE] Age-Linear x Match: −0.555±0.175; t_690_ = −3.167, p = 0.002). However, in the second phase, i.e. between days 8–15, their growth curve was more strongly inflected (Age-Quadratic x Match: 0.380±0.122; t_690_ = −3.111, p = 0.002), and consequently nestling body mass was similar to that of nestlings from mothers exposed to high prenatal predation risk under mismatch (Tukey adjusted *post hoc*: *p* = 0.259). In contrast, comparing growth trajectories of nestlings from mothers exposed to the prenatal control treatment in matching or mismatching conditions showed no difference in curvature (i.e. no change in mass-gain rate; Age-Linear x Mismatch [coef. ± SE]: 0.376±0.145; t_624_ = 2.595, p = 0.010; Age-Quadratic x Mismatch: −0.018±0.104; t_624_ = −0.168, p = 0.866). Nestling mass on day 15 did not differ among the four treatment combinations groups (all p>0.2).

**Table 3:** ANOVA table and estimated coefficients – linear mixed model for nestling mass.

Variable	Estimate (SE)	df	*F*	*p*
Intercept	9.510 (0.157)	–	–-	–-
Brood size	0.087 (0.042)	1,106	4.175	0.044
Hatching date	−0.025 (0.014)	1,106	3.421	0.067
Sex^a^	−0.001 (0.033)	1,548	0.001	0.975
Age		2,1314	3061.438	<0.001
Predator treatment	0.548 (0.215)	1,106	6.466	0.012
Match treatment	0.144 (0.224)	1,106	0.413	0.522
Age x Predator		1,1314	4.456	0.012
Age x Match		2,1314	2.541	0.079
Predator x Match	−0.672 (0.306)	1,106	4.810	0.031
Age x Predator x Match		2,1314	10.689	<0.001
Estimates for coefficients of growth curves:				
Treatment group	Linear	Quadratic		
Prenatal control - match	9.072 (0.116)	−0.556 (0.082)		
Prenatal control - mismatch	9.447 (0.120)	−0.574 (0.084)		
Prenatal predator - match	0.484 (0.112)	−0.747 (0.079)		
Prenatal predator - mismatch	8.929 (0.112)	−0.367 (0.079)		

A repeated measurements model with nestling identification nested within Nest of Origin as random factors. Age was taken as an ordered categorical factor. Linear and Quadratic coefficient estimates of age are provided for each treatment group. Hatching date and brood size are centred for ease of interpretation. *F* and *p* values originate from the ANOVA table.

SE  =  standard error.

a For a male compared to a female.

An analysis of mass gain in the different growth phases indeed shows a significant interaction between the prenatal maternal and the match treatments for mass gain in the fast growth phase, i.e. days 2–8 (*F*_1,106_ = 5.969, *p* = 0.016), with more mass gained by nestlings of mothers exposed prenatally to increased predation risk when in a matching environment than when in a mismatch environment (Tukey adj. *post hoc*: *p* = 0.028). However, we found no differences in mass gain between days 8–15 (interaction term: *F*_1,106_ = 0.611, *p* = 0.436).

The effects of the two treatments on skeletal size, represented by tarsus length, depended on the treatment level combination (significant interaction predator x match treatment; Table 4). A mismatch between the prenatal maternal environment and the postnatal environment had a positive effect for nestlings of the control group, but a negative effect for offspring of the predator group. *Post-hoc* comparisons between the 4 groups on both day 8 and 15 revealed no significant differences between any of the groups (all comparisons *p*>0.12).

**Table 4:** ANOVA table and estimated coefficients – linear mixed model for nestling morphological traits.

Measurement	Variable	Estimate (SE)	df	*F*	*p*
Tarsus	Intercept	12.653 (0.155)	–	–	–
	Brood size on day 2	−0.259 (0.058)	1,106	19.804	<0.001
	Hatching date	0.071 (0.019)	1,106	14.441	<0.001
	Sex	0.336 (0.066)	1,548	25.870	<0.001
	Age	6.250 (0.053)	1,659	14113.742	<0.001
	Predator treatment	0.297 (0.204)	1,106	2.134	0.147
	Match treatment	0.273 (0.215)	1,106	1.615	0.207
	Predator x Age	−0.173 (0.105)	1,658	2.718	0.100
	Match x Age	−0.100 (0.108)	1,657	0.870	0.351
	Predator x Match	−0.610 (0.293)	1,106	4.325	0.040
	Predator x Match x Age	0.282 (0.215)	1,656	1.308	0.191
Wing	Intercept	18.019 (0.520)	–	–	–
	Brood size on day 2	−0.752 (0.192)	1,106	15.235	<0.001
	Hatching date	0.324 (0.062)	1,106	27.777	<0.001
	Sex	0.494 (0.220)	1,548	5.059	0.025
	Age	28.377 (0.222)	1,657	16360.233	<0.001
	Predator treatment	0.804 (0.697)	1,106	1.332	0.251
	Match treatment	0.320 (0.730)	1,106	0.193	0.662
	Predator x Age	0.173 (0.311)	1,657	0.307	0.580
	Match x Age	0.720 (0.313)	1,657	5.276	0.022
	Predator x Match	−1.536 (1.001)	1,106	2.354	0.128
	Predator x Match x Age	−1.075 (0.487)	1,657	4.864	0.028

Wing and tarsus models are repeated measures models with Nest of Origin and nestling ID as random factors. Hatching date and brood size are centred for ease of interpretation. *F* and *p* values stem from ANOVA table, and for non-significant interactions represent values just before removal (significance level for removal of interaction *p*>0.1). Reference  = 8 days old female nestling from a prenatal maternal control and matching environment group. SE  =  standard error.

Wing growth showed different rates according to the treatment combinations (significant interaction Predator x Match x Age; Table 4). Comparing slopes by fixating each maternal treatment at a time revealed that control group nestlings grew wings faster when in a mismatching environment than when in a matching environment (Age x Match estimate ± SE = 0.72±0.32; *t*_312_ = 2.283, *p* = 0.023). Growth rate did not differ with respect to environment for predator group nestlings (*t*_345_ = −0.959, *p* = 0.338). *Post hoc* tests on day 15 showed that nestlings originating from predator-treated mothers had marginally smaller wings when in a mismatch environment (Match-Mismatch: estimate±SE = −2.35±0.92, *p* = 0.055). On day 8 the difference was in the same direction, and significant (Match-Mismatch: estimate ± SE = −1.89±0.03, *p* = 0.031).

Parental feeding rate did not differ between the treatments, nor was it influenced by the interaction between the two treatments (Theta for negative-binomial distribution 3.902, all *p*>0.3). There was a slight increase in feeding rate over the breeding season (estimate ± SE: 0.049±0.012, *χ* ^2^ = 16.291, *p*<0.001). Within pairs, males fed in average more than females ([mean ± SD] males: 19.55±11.12; females: 14.82±9.74; paired *t*-test: *t*_96_ = −5.3795, *p*<0.001).

## Discussion

Whether or not the role of a specific maternal effect is adaptive is not always straightforward [31]. Even when expected to be adaptive, identifying the main benefiter may prove difficult, for example if the maternal effect consists of the facilitation of brood reduction in order to increase female lifetime reproductive success at the expense of current offspring [11], [16]. In addition, identifying the incurred benefits of a maternal effect may be difficult since these may become apparent at later life-stages only [31]. To further complicate matters, when a maternal effect is targeted to a specific environment, a mismatch of the maternal and offspring environments may not only conceal the adaptive value of the effect, but rebound and become costly for the offspring, e.g. [32].

Here, by manipulating both the prenatal maternal environment and the postnatal environment to match or mismatch, we could both identify the presence of a maternal effect and get an idea of its intended benefiters. The growth of offspring from mothers exposed to increased prenatal predation risk depended on the matching of the prenatal maternal and postnatal environments. Although the growth of nestlings from control mothers was, if anything, slightly faster when growing under mismatching environments, i.e. high predation risk, for nestlings of predation-exposed mothers growth was reduced under mismatching conditions. We found differences in the shape of growth curves: when growing in a mismatching environment, nestlings of mothers under prenatal increased predation risk gained less mass during the fast-growth stage (days 2–8) compared to those in a matching environment. This could be due to a delay in the start of the rapid growth phase. The opposite was true for nestlings of the prenatal control group. Although there were no significant differences in mass of 15 days old nestlings, this suggests that nestlings of the prenatal predator group in a mismatch environment had reached their asymptotic mass, typically reached 12–14 days after hatching [20], [21], later than those under matching conditions. If mass gain in predator-mismatch nestlings simply started later and was then more rapid than in matching nestlings, this could be taken as evidence for catch-up growth [33]. However, our three measurement points prevent us from determining the exact shape of the growth curves and hence from taking this conclusion. Alternatively, reaching asymptotic mass later could be indicative for a prolonged growth period, which has been suggested as another mechanism of compensatory growth, e.g. [34], [35]. This idea is supported by the increase of almost one full day in fledging age of nestlings of prenatal predator-treated mothers when under mismatching conditions. A prolonged growth period may put nestlings at a disadvantage after fledging when competing with conspecifics fledged at the optimal age. In addition, a longer nestling phase could increase the likelihood of nest predation, the most important source of reproductive failure for many birds [2], [36], and may also increase parental effort and thus influence parental trade-offs.

Several studies suggest that a minimal wing length is required for fledging, e.g. [37], [38]. Nestlings of the predator group did not differ in the rate of wing growth between days 8–15, but under mismatching conditions had shorter wings on both days. Wings start developing in the great tit around day 8 after hatching [20], [21]. Growing slower at an early growth stage, as suggested by the mass growth curves, may have caused nestlings of the prenatal predator group under a mismatching environment to start developing wings later. Thus, with no acceleration of wing growth these nestlings would reach the required wing length for fledging later, which may also explain their later fledging time. The effect on final wing length is not clear from these results alone, however, since on day 15 nestling wings are only about 2/3 of their final length in the great tit, and continue to grow after fledging [20], [21]. Given that clutch size did not differ between the treatments, and that neither treatment nor the interaction between the two significantly affected fledging success or parental effort (when testing feeding rates), it suggests that the maternal effects observed on nestling morphology were aimed at adjusting offspring phenotype to local conditions. Such ‘anticipatory maternal effects’ [6] increase maternal fitness by increasing offspring fitness, for example by increasing survival probability. However, to increase offspring fitness, the offspring environment cannot drastically differ from that of mothers. Thus, although maternal effects may evolve as a plastic response to differing environmental conditions, such ‘anticipatory maternal effects’ are expected to evolve when environmental conditions vary but are predictable in the short term [6], [19]. In this experiment, the effects of changing postnatal conditions to match or not to match prenatal maternal ones depended on the conditions mothers experienced. Whereas for offspring of prenatal control mothers the effect of a mismatch in the environmental conditions was generally not significant or rather positive, for offspring of females ovulating during increased perceived predation risk the mismatch generally had a negative effect. This suggests a cost for growth in nestlings of mothers that predicted environmental conditions wrongly. It appears that this growth cost comes into effect only once the maternal effect is induced. The cost of mismatch if not inducing a maternal effect (e.g. our control-mismatch group) would be reduced offspring survival or fitness in comparison to conspecifics prepared for specific environmental challenges, although we lack the data to confirm these suggestions.

Such costs resemble mismatch costs as suggested for the ‘thrifty phenotype’, reviewed by [31], and the ‘weather forecast’ model suggested by Bateson *et al*. [18]. Under these hypotheses, costs associated with developing of the thrifty or the ‘forecasted’ phenotype are paid when the offspring is invoked to develop a phenotype that maximizes its fitness under specific demanding conditions but eventually faces relaxed conditions. For example, humans experiencing poor nutritional conditions *in utero* but growing later under good feeding conditions suffer increased risk of diseases such as type-2 diabetes and obesity [31], [39]. In this case the poor performance of a phenotype is based on the environmental mismatch and not on trade-offs [31]. Since life-histories of many organisms involve periods (often brief) sensitive to environmental triggers, such as early growth [18], the costs of such mismatches may be irreversible, in particular in species showing determinate growth. This makes the match between the prenatal maternal environment and the environment of the developing offspring, in our case in the nestling postnatal period, extremely important. The resemblance of our results to the ‘weather forecast’ model [18] is enhanced by the fact that the cost of mismatch was only present when the maternal effect was induced, that is under increased prenatal perceived predation risk for mothers. The costs of mismatch when not inducing a specific phenotype via a maternal effect would appear later via reduced survival in a predator-rich environment.

The proximate mechanisms leading to the observed costs of mismatch are unknown. Increases in nestling stress, and stress hormone levels, due to the presence of predators in the environment, may have effects on growth that could differ according to maternal preparation. Additionally, changes in parental provisioning may interact with the maternal preparation of the nestlings. Parental feeding may change without changing the frequency of feeding bouts but rather the frequency of prey types provided to nestlings, e.g. [40]. In another experiment we found that under increased perceived predation risk by sparrowhawks, parents reduced their selectivity of prey types compared to control parents [41]. Maternal effects in birds can be invoked via different mechanisms. Known examples include deposition of antibodies [42], antioxidants [43], and different hormones such as androgens and glucocorticoids [17], [44] in the eggs. Behavioral mechanisms such as alteration of incubation behavior [45] may also be affected by risk of predation and possibly influence offspring phenotype. Since we found no difference in egg mass between the treatments, the maternal effect must have been conveyed through egg content or incubation behaviors. In another study [46] we found that great tit females exposed to predation risk deposited lower levels of testosterone in eggs, supporting the possibility of a hormonal mechanism.

To summarize, the results suggest that an increase in perceived predation risk before and during ovulation of great tit females induced a maternal effect that affected offspring growth. Given that the influence of this maternal effect depended on the rearing environment of the nestlings and may also provide benefits to offspring [7], it suggests that the maternal effect is adaptive. The potentially adaptive effect does not seem to arise via influencing the trade-off between current and future reproduction by facilitation of brood reduction, but rather by directly influencing offspring phenotype, aiming to increase the fit of offspring to the environment. Triggering these maternal effects may incur costs for nestling growth when the environment does not match expected conditions later. Our results are relevant to a mismatch of the prenatal maternal and the postnatal environments and are based on known correlations between size and mass at fledging with respect to future survival prospects of birds, e.g. [12]. To further conclude on the cost of mismatch in the environments it would be necessary to compare the reproductive success of nestlings both growing and living until recruitment in matching or mismatching environments, which was beyond the scope of this study.

## Supporting Information

Table S1 **Data summary by maternal treatment groups.**
(DOCX)

## References

[1] Caro T (2005) Antipredator defenses in birds and mammals. Chicago: University of Chicago Press.

[2] Lima SL (2009) Predators and the breeding bird: behavioral and reproductive flexibility under the risk of predation. Biological Reviews 84: 485–513. 19659887 10.1111/j.1469-185X.2009.00085.x

[3] Martin TE, Briskie JV (2009) Predation on Dependent Offspring. Annals of the New York Academy of Sciences 1168: 201–217. 19566709 10.1111/j.1749-6632.2009.04577.x

[4] Remes V, Martin TE (2002) Environmental influences on the evolution of growth and developmental rates in passerines. Evolution 56: 2505–2518. 12583590 10.1111/j.0014-3820.2002.tb00175.x

[5] Mousseau TA, Fox CW (1998) The adaptive significance of maternal effects. Trends in Ecology & Evolution 13: 403–407. 21238360 10.1016/s0169-5347(98)01472-4

[6] Marshall DJ, Uller T (2007) When is a maternal effect adaptive? Oikos 116: 1957–1963.

[7] Coslovsky M, Richner H (2011) Predation risk affects offspring growth via maternal effects. Functional Ecology 25: 878–888.

[8] Giesing ER, Suski CD, Warner RE, Bell AM (2011) Female sticklebacks transfer information via eggs: effects of maternal experience with predators on offspring. Proceedings of the Royal Society B: Biological Sciences 278: 1753–1759. 21068041 10.1098/rspb.2010.1819 PMC3081764

[9] Storm JJ, Lima SL (2010) Mothers Forewarn Offspring about Predators: A Transgenerational Maternal Effect on Behavior. The American Naturalist 175: 382–390. 10.1086/650443 20109061

[10] Saino N, Romano M, Ferrari RP, Martinelli R, Moller AP (2005) Stressed mothers lay eggs with high corticosterone levels which produce low-quality offspring. Journal of Experimental Zoology Part a-Comparative Experimental Biology 303A: 998–1006. 10.1002/jez.a.224 16217808

[11] Hayward LS, Wingfield JC (2004) Maternal corticosterone is transferred to avian yolk and may alter offspring growth and adult phenotype. General and Comparative Endocrinology 135: 365–371. 14723888 10.1016/j.ygcen.2003.11.002

[12] Naef-Daenzer B, Widmer F, Nuber M (2001) Differential post-fledging survival of great and coal tits in relation to their condition and fledging date. Journal of Animal Ecology 70: 730–738.

[13] Rubolini D, Romano M, Boncoraglio G, Ferrari RP, Martinelli R, et al (2005) Effects of elevated egg corticosterone levels on behavior, growth, and immunity of yellow-legged gull (Larus michahellis) chicks. Hormones and Behavior 47: 592–605. 15811362 10.1016/j.yhbeh.2005.01.006

[14] Witter MS, Cuthill IC, Bonser RHC (1994) Experimental investigations of mass-dependent predation risk in the European starling, Sturnus vulgaris. Animal Behaviour 48: 201–222.

[15] Chin EH, Love OP, Verspoor JJ, Williams TD, Rowley K, et al (2009) Juveniles exposed to embryonic corticosterone have enhanced flight performance. Proceedings of the Royal Society B: Biological Sciences 276: 499–505. 18842541 10.1098/rspb.2008.1294 PMC2664354

[16] Love OP, Williams TD (2008) The Adaptive Value of Stress-Induced Phenotypes: Effects of Maternally Derived Corticosterone on Sex-Biased Investment, Cost of Reproduction, and Maternal Fitness. The American Naturalist 172: E135–E149. 10.1086/590959 18793091

[17] Henriksen R, Rettenbacher S, Groothuis TGG (2011) Prenatal stress in birds: Pathways, effects, function and perspectives. Neuroscience & Biobehavioral Reviews 35: 1484–1501. 21536067 10.1016/j.neubiorev.2011.04.010

[18] Bateson P, Barker D, Clutton-Brock T, Deb D, D’Udine B, et al (2004) Developmental plasticity and human health. Nature 430: 419–421. 15269759 10.1038/nature02725

[19] Wells JCK (2007) Environmental Quality, Developmental Plasticity and the Thrifty Phenotype: A Review of Evolutionary Models. Evolutionary Bioinformatics 2007. PMC2684123 19461971

[20] Gosler A (1993) The Great tit; Christie D, editor. London: Hamlyn.

[21] Perrins CM (1979) British tits; Davies M, Gilmour J, Mellanby K, editors. Glasgow: William Collins Sons & Co.

[22] Verhulst S, Nilsson J-Å (2008) The timing of birds’ breeding seasons: a review of experiments that manipulated timing of breeding. Philosophical Transactions of the Royal Society B: Biological Sciences 363: 399–410. 10.1098/rstb.2007.2146 PMC2606757 17666390

[23] Griffiths R, Double MC, Orr K, Dawson RJG (1998) A DNA test to sex most birds. Molecular Ecology 7: 1071–1075. 9711866 10.1046/j.1365-294x.1998.00389.x

[24] R DC (2010) R: A language and environment for statistical computing. 2.12.0 ed. Vienna, Austria.

[25] Pinheiro JC, Bates DM, DebRoy S, Deepayan S, team RDC (2012) nlme: Linear and Nonlinear Mixed Effects Models. R package version 3.1–103 ed.

[26] Bates D, Maechler M, Dai B (2008) lme4: Linear mixed-effects models using S4 classes. R package version 0.999375–28 ed.

[27] Pinheiro JC, Bates DM (2000) Mixed-effects models in S and S-Plus; Chambers J, Eddy W, Hardle W, Sheather S, Tierney L, editors. New York: Springer Verlag.

[28] Hothorn T, Bretz F, Westfall P (2008) Simultaneous Inference in General Parametric Models. Biometrical Journal 50: 346–363. 18481363 10.1002/bimj.200810425

[29] Venables WN, Ripley BD (2002) Modern Applied Statistics with S. New York: Springer.

[30] Engqvist L (2005) The mistreatment of covariate interaction terms in linear model analyses of behavioural and evolutionary ecology studies. Animal Behaviour 70: 967–971.

[31] Monaghan P (2008) Early growth conditions, phenotypic development and environmental change. Philosophical Transactions of the Royal Society B: Biological Sciences 363: 1635–1645. 10.1098/rstb.2007.0011 PMC2606729 18048301

[32] Love OP, Williams TD (2008) Plasticity in the adrenocortical response of a free-living vertebrate: The role of pre- and post-natal developmental stress. Hormones and Behavior 54: 496–505. 18313054 10.1016/j.yhbeh.2008.01.006

[33] Metcalfe NB, Monaghan P (2001) Compensation for a bad start: grow now, pay later? Trends in Ecology & Evolution 16: 254–260. 11301155 10.1016/s0169-5347(01)02124-3

[34] Bize P, Roulin A, Bersier LF, Pfluger D, Richner H (2003) Parasitism and developmental plasticity in Alpine swift nestlings. Journal of Animal Ecology 72: 633–639. 10.1046/j.1365-2656.2003.00734.x 30893964

[35] Emlen ST, Wrege PH, Demong NJ, Hegner RE (1991) Flexible Growth Rates in Nestling White-Fronted Bee-Eaters: A Possible Adaptation to Short-Term Food Shortage. The Condor 93: 591–597.

[36] Ricklefs RE (1969) Natural selection and development of mortality rates in young birds. Nature 223: 922-&. 10.1038/223922a0 5803396

[37] Nilsson J-A, Svensson M (1996) Sibling Competition Affects Nestling Growth Strategies in Marsh Tits. Journal of Animal Ecology 65: 825–836.

[38] Bize P, Metcalfe NB, Roulin A (2006) Catch-up growth strategies differ between body structures: interactions between age and structure-specific growth in wild nestling Alpine Swifts. Functional Ecology 20: 857–864.

[39] Wells JCK (2007) The thrifty phenotype as an adaptive maternal effect. Biological Reviews 82: 143–172. 17313527 10.1111/j.1469-185X.2006.00007.x

[40] Tarwater CE, Kelley JP, Brawn JD (2009) Parental response to elevated begging in a high predation, tropical environment. Animal Behaviour 78: 1239–1245.

[41] Coslovsky M, Richner H (2012) An experimental test of predator-parasite interaction in a Passerine bird. Oikos *in press*.

[42] Grindstaff JL, Brodie ED, Ketterson ED (2003) Immune function across generations: integrating mechanism and evolutionary process in maternal antibody transmission. Proceedings of the Royal Society of London Series B: Biological Sciences 270: 2309–2319. 14667346 10.1098/rspb.2003.2485 PMC1691520

[43] Berthouly A, Helfenstein F, Richner H (2007) Cellular immune response, stress resistance and competitiveness in nestling great tits in relation to maternally transmitted carotenoids. Functional Ecology 21: 335–343.

[44] Groothuis TGG, Müller W, von Engelhardt N, Carere C, Eising C (2005) Maternal hormones as a tool to adjust offspring phenotype in avian species. Neuroscience & Biobehavioral Reviews 29: 329–352. 15811503 10.1016/j.neubiorev.2004.12.002

[45] Kovařík P, Pavel V (2011) Does Threat to the Nest Affect Incubation Rhythm in a Small Passerine? Ethology 117: 181–187.

[46] Coslovsky M, Groothuis T, de Vries B, Richner H (2012) Maternal steroids in egg yolk as a pathway to translate predation risk to offspring: Experiments with great tits. General and Comparative Endocrinology. 10.1016/j.ygcen.2012.01.013 22326354

