# Supplementary material for: Preparing Offspring for a Dangerous World: Potential Costs of Being Wrong
Source: PLoS One. 2012 Nov 7;7(11):e48840. doi: 10.1371/journal.pone.0048840 (PMC3492257; doi:10.1371/journal.pone.0048840)
Supplement: Table S1 — Data summary by maternal treatment groups. (DOCX) [file pone.0048840.s001.docx]

Table S1 Data summary by maternal treatment groups

|  | Control | Predator |
| --- | --- | --- |
| Treatment time (days) | 37.22 ± 5.17 | 38.37 ± 4.42 |
| Laying date | 29.32 ± 4.85 | 30.03 ± 5.33 |
| Clutch size | 8.14 ± 1.87 | 8.23 ± 1.73 |
| Incubation time (days) | 11.95 ± 1.24 | 11.87 ± 1.10 |
| Hatching date | 50.71 ± 4.46 | 50.83 ± 4.34 |
| Brood size at hatching | 7.25 ± 1.41 | 7.00 ± 1.34 |
| Mean egg mass (g) | 1.58 ± 0.10 | 1.60 ± 0.13 |

Values are mean ± SD. Laying/hatching dates take 15.3.09 as 0. Number of nestlings is on 2^nd^ day after hatching. Treatment time is from the simulations begin until start of egg-laying.
